# Guidelines for Research Data Integrity (GRDI)

**DOI:** 10.1038/s41597-024-04312-x

**Published:** 2025-01-17

**Authors:** Gregor Miller, Elmar Spiegel

**Affiliations:** Core Facility Statistical Consulting, Helmholtz Zentrum München, 85764 Neuherberg, Germany

**Keywords:** Research data, Computational biology and bioinformatics, Medical research, Research management

## Abstract

Ensuring the integrity of research data is crucial for the accuracy and reproducibility of any data-based scientific study. This can only be achieved by establishing and implementing strict rules for the handling of research data. Essential steps for achieving high-quality data involve planning what data to gather, collecting it in the correct manner, and processing it in a robust and reproducible way. Despite its importance, a comprehensive framework detailing how to achieve data quality is currently unavailable. To address this gap, our study proposes guidelines designed to establish a reliable approach to data handling. They provide clear and practical instructions for the complete research process, including an overall data collection strategy, variable definitions, and data processing recommendations. In addition to raising awareness about potential pitfalls and establishing standardization in research data usage, the proposed guidelines serve as a reference for researchers to provide a consistent standard of data quality. Furthermore, they improve the robustness and reliability of the scientific landscape by emphasising the critical role of data quality in research.

## Introduction

A crisis of reproducibility in scientific research has long been identified^1^ and most researchers are aware of it^2^. This issue undermines the trustworthiness of science, and various measures have been taken to mitigate the problem. An increasing number of journals require authors to follow reporting guidelines, which are continuously updated, to ensure that studies are described in a standardized and comprehensive manner^3–5^. In recent years, important efforts have been made to enhance the impact of data collection. This includes the FAIR principles, which aim to make data more machine-readable, findable, and reusable^6^. The FAIR principles and other initiatives intend to promote data accessibility in an open and responsible manner, as well as data sharing among researchers^7^. Major funding agencies have published their own guidelines for data management and now require data management plans^8–10^.

Despite these efforts, the number of retractions is continuously increasing^11^. While the detailed reasons for retractions, and their origin as either honest mistakes or malicious mishandling, are often unclear, some reasons have been reported. These include wrong labelling^12–15^, the use of the wrong dataset^16,17^, duplication of entries^18,19^, discrepancies in the data^19–21^, and coding errors^21^. In some cases it became clear that unsuitable software was used^12,22^. It is possible that erroneous data handling stays unnoticed, resulting in an unknown number of publications being based on such errors. This issue has already been suggested by previous publications^23,24^.

However, efforts to improve data management and sharing frequently concentrate on data transferability and infrastructure, or on the provision of administrative guidelines. While these are important for enabling future data use by external researchers, they do not address issues like data integrity or data processing during analysis. Proper structuring and processing of data are crucial cornerstones of scientific research. This also helps to avoid mistakes in data handling and makes it more difficult to manipulate data to produce more suitable results. Reliable and reproducible research requires robust data gathering and pre-processing before any analyses are conducted. The quality of the data will heavily impact the analysis results. It is important to note that the sharing of data, although valuable, is not a sufficient indicator of the quality of data processing.

The main aim of this publication is to provide any researcher, who is involved in the data collection and handling process at some point, with practical and clear guidelines for handling and processing their data. Additionally, we aim to emphasise the importance of these processes and encourage critical discussion about prioritising the integrity of study data. The guidelines are based on years of experience in working with data analysis projects from various scientific fields including medicine, psychology, and natural sciences.

The main principles that underpin the guidelines are outlined first, followed by the guidelines organised according to the typical research project process. The research process typically begins with planning, followed by data collection or a request for data from a repository, and concludes with data handling during the analysis phase.

## Principles

Scientific data handling is important in all research fields where data is collected and analysed. However, each field has its own unique data and requirements, so guidelines for implementing robust data handling cannot be comprehensive or cover all possible scenarios. The following principles can help identify issues that need to be considered and resolve conflicts.

1. Accuracy - Does the data accurately represent what is observed? It may seem trivial, but machine inaccuracy, incorrect operation of machinery, incorrect input, wrong nomenclature, and erroneous variable transformation can challenge this presumption and may not be obvious to detect.
2. Completeness – Does the data contain enough relevant information? In addition to primary variables, other information may need to be collected. This can relate to confounders that change the behaviour of other variables, changes in the data collection process, or identifiability of entries.
3. Reproducibility - Can the data collection and processing be reproduced? This extends the completeness principle by metainformation of the dataset and its processing. This may involve reporting data versions, specifics of database queries, or changes in the data itself.
4. Understandability - Can a layperson understand the data or does it require specific knowledge of the domain, language, or programming? The more people who can understand the data, the easier it is to understand derived results and use or reproduce the data.
5. Interpretability - Can everyone draw the right conclusions from the data? If the data is comprehensible but prone to misinterpretation, there is a high risk of false confidence in its use and the results.
6. Transferability - Can the data be read without errors using different software? Similarly to understandability, transferability enhances the power of the dataset.

It is evident that these principles may occasionally conflict. As instance, while the completeness of a dataset increases with the amount of information it contains, the accuracy becomes more challenging due to potential input errors. Therefore, balancing these principles can be challenging. The following guidelines aim to assist researchers by providing practical advice on how to adhere to these principles in a proven approach.

## Guidelines

The guidelines are organised according to the typical research process. While some may be more important than others depending on the type of study, data, and research field, all are relevant and should be considered. The first four sections focus on data collection and acquisition, while the last section is dedicated to processing the available data. Depending on the project, different aspects may need to be addressed by different researchers or by a single researcher. A practical summary of the guidelines in checklist format can be found in the supplementary material and the Gitlab repository at https://ascgitlab.helmholtz-munich.de/cf_statcon/GRDI.

### Defining the strategy

The general strategy of a study determines the objectives and main elements of the study. These broad specifications need to be defined and discussed before detailed preparations are made, otherwise major revisions of the data collection and handling process are likely.

#### Plan study, data requirements, and analysis together

The study’s objective determines what needs to be measured, the available data determines what can be analysed, and the type of analysis determines the conclusion that can be drawn. Therefore, it is crucial to plan all three steps together and define the requirements necessary to complete each step. An example for different objectives is data collected for diabetes research which has limited usability for nutrition-allergy reports.

#### Write a data dictionary

In order to ensure interpretability, it is recommended to write a clear and concise data dictionary in a separate file. This dictionary should explain all variable names, the coding of their categories^25,26^, and their units as well as provide context for the data such as the time, reason, and method of its collection. For example, the categories of the education level of participants might be coded as “0 = no formal education”, “1 = high school diploma”, “2 = Bachelor’s degree” and so on.

The dictionary should be prepared before and completed during data collection to ensure prompt identification of issues for example with respect to category levels, machine precision, measurement units, expected range of values etc. This should also include rules that were used to validate the entered data. Adding metadata, including the objective for the data collection, measurement techniques, and modifications to the methodology, into a dictionary or separate document can facilitate the meaningful use of the data by ensuring accessibility, understanding, and appropriate interpretation and processing^27^. Where available, it is recommended that controlled vocabularies are used to standardise data and metadata terms, thereby ensuring comparability. Furthermore, the dictionary should remain closely linked to the data to prevent loss or outdated information.

#### Save your data in an accessible and general-purpose file format

To ensure accessibility over time and across computing systems, it is important to save data in a suitable file format. This includes for example CSV files for tabular data or XML format for structured information, and in general will depend on what kind of data is being saved^25,27,28^. If there are multiple suitable formats, it is advisable to choose the one that is most commonly used in the respective research field because it is more likely to be supported by the community and have well-established tools for analysis and sharing. It can be helpful to review the requirements of open repositories and organisations that set standards for data sharing, such as the Research Data Alliance, to determine what format is suitable. However, it is crucial to assess whether the format is open and sufficiently supported by open-source software. If a widely used format is not open, researchers should still consider its community support and the availability of conversion tools but prioritise open and platform-independent formats for long-term accessibility.

#### Keep the raw data

Generally, raw data represents the data in its most unaltered form. This can mean raw sensor outputs in experimental sciences, unedited survey responses, or raw medical images. It is important to save the raw, unprocessed data, preferably in multiple locations, even if only processed data is being worked with^25–27^. This is necessary in case changes to the processing are needed or when merging with data from other sources. This can also apply to backups which are created while the data is being collected. In the case of multiple versions of the data, it is crucial to maintain a consistent version that is worked on to avoid confusion. If a versioning system is not used, at least define the date or version number for each file manually.

### Defining all variables

Once the general questions and goals of the data gathering have been determined, it is necessary to specify the collection process. Determining how variables are stored, including their definition, structure, and type, helps to prevent errors in the data collection process, improves clarity, and ensures that all necessary information is available for later analysis.

#### Avoid repetition

Unnecessary repetition of equivalent or similar inputs increases the risk of incorrect entries and inconsistencies. Manual data collection is often done in a repetitive and stressful environment where it may be hard to focus on accuracy, therefore, it is advisable to reduce the effort of entering data and avoid creating separate entries for potentially similar inputs. Transforming variables into new units or coding can be done efficiently, accurately, and in a reproducible manner by any script program later in the processing stage. For example, defining the exact diagnosis of a patient but then also adding the higher-level classification of the diagnosis is unnecessary and can easily be done when processing the data.

#### Avoid combining information

Joining information is typically straightforward, whereas separating information can be challenging or even impossible. For instance, consider a person’s first and last name. Different cultures have varying numbers of names, making it difficult to separate them manually or programmatically, particularly when dealing with large datasets from different sources. It is important to avoid combining information when it comes to numbers, as retrieving the original raw numbers may be impossible, which could be crucial for future processing and analysis. In general, it is advisable to retain the non-scaled, non-transformed variables and absolute values rather than relative values. While the use of relative values may be appropriate for data presentation and the communication of findings, it can also be problematic in cases where the precise calculation of data ratios is lost. Therefore, it is advisable that the raw absolute values are stored. The combination, transformation, and aggregation of information is done later in the data processing using scripts to improve accuracy and reproducibility.

#### Use simple language

Use clear and concise language without sacrificing precision in names and statements. Especially the data dictionary should be as understandable as possible. This will support data collectors in gathering the data and allow researchers from diverse backgrounds and non-native speakers to access the data.

#### Give short but informative variable names

Using “diabetes_type” instead of “diab” for a variable improves interpretability and clarifies its meaning. In general, variable names should be self-explanatory and unambiguous^25,28^. For example, “name” could refer to either the first or last name of a person. Long variable names can be unwieldy and tedious to work with during processing. Therefore, it is necessary to find a good compromise for their length. Comparing the used variable names with domain-specific naming conventions prevents ambiguity and misinterpretation of data.

#### Make column names machine-readable

When naming variables in your data, use only basic letters, numbers, and underscores. This means avoiding spaces, commas, semicolons, umlauts, or other special symbols^28^. Although some programs may allow the use of such symbols, it can prevent other programs from being used or affect transferability. Therefore, “age_in_years” is preferable as column name to “age [years]”.

#### Record metadata

Comprehensive data descriptions provide contextual information necessary for informed decision-making during data processing and analysis. This includes stating the general goal and specific hypotheses for data collection as well as details on the data collection process, including any changes made to hardware, experimenters, or dataset structure. Additionally, providing explanations for missing values, variable names, and labels is crucial for ensuring the usability of the data^9^. Adopting a widely accepted metadata standard, such as Dublin Core, is recommended for ensuring consistency and interoperability^29^.

#### Use a suitable tool for data capture, management, and storage

While spreadsheets are readily available and easy to set up, they have significant limitations for data management. This includes restricted data entry control, potential for data entry errors, and issues with automatic formatting leading to misinterpretation, such as with dates and gene names^12^. Additionally, they allow saving information in inaccessible ways, such as through colour or field calculations. To ensure robust and reliable data collection, it is recommended to use a specialized data collection system instead.

A variety of data collection systems are available^30^. The choice of an appropriate system depends on a number of factors, including licensing, importing and exporting capabilities and specific study requirements. Therefore, it is necessary to compare available collection systems and the needs of the study. With this system data should be digitalized as early as possible to minimize human involvement in data entry and focus resources on ensuring data quality and flow.

#### Account for varying levels of measurement accuracy among entries

Variations in accuracy can be attributed to a range of factors, such as the use of devices with different accuracy limits, or the fact that, for example, one hospital only records the presence of diabetes, while another distinguishes between type 1 and type 2 diabetes. The first challenge is to be aware of such differences, and the second is to analyse the data together. Awareness is achieved by creating detailed documentation and maintaining close communication between all data gathering entities. The method for analysing the data together depends on the type of data. In some cases, it may be sufficient to transform the accuracy of all datasets to a common level, while in others, the analysis must account for these differences.

### Defining the collection process

Once the relevant variables have been defined, it is important to also establish the structure and type of the entries themselves. This involves determining what should be entered for each variable including consistent categories and clear definitions for missing values.

#### Use identifiers

Each sample or subject should be assigned a unique identifier from the start. Typically, an integer is used as the identifier since other information is often not unique on its own. This identifier should be assigned at the level of each sample, subject, or experimental unit. For example, a patient whose medical values were measured multiple times always gets the same identifying number. Using unique identifiers ensures that every value entered is clearly attributed to the corresponding identifier, making duplicated entries easily findable and new information can be attributed unambiguously to the correct sample.

When dealing with patient data, it is important to consider de-identification strategies prior to data dissemination. This might involve binning or grouping sensitive values (e.g., age ranges instead of exact ages) and ensuring that identifiers do not inadvertently reveal personal information.

#### Be concise but avoid ambiguity

When entering a value of “1” into a “treatment” column, it is unclear whether the patient was treated or not, or if they received the first treatment. To avoid confusion, categorical entries should have meaningful codes. In this case, using “Untreated” and “Treated” as categories would make the options intuitively clear and prevent errors.

#### Restrict data entry to possible values

When collecting data, only meaningful values should be allowed. For instance, negative values for height measurements are not sensible. If there are predefined categories, only those categories should be allowed to be entered using a predefined naming scheme.

#### Be consistent with nomenclature

Prior to the start of data collection, a consistent and clear nomenclature needs to be established. Consequently, categorical values should always get the same coding, the same variable in different tables should have the same name, and all cells within a column should contain only one data type, meaning only numeric, categorical, or date values^27^. But also file names, dates, and data layout should follow a consistent format^27,28^. Switching months and days in a date variable would be a simple mistake but can render a variable or many observations unusable as it is unclear where the respective scheme has been used. For dates and times, it is advisable to use the formats “yyyy-mm-dd” and “hh:mm” with a 24-hour-clock system. For example, this would mean using “2023-05-14” instead of “05/14/2023” and “14:05” instead of “2:05 pm”.

Even floating-point numerical values can bear the risk of misinterpretation, in particular when they are shown in different, region-specific formats. A value of “11.362” might be in interpreted in Europe as 11 362 (eleven-thousand and three hundred sixty-two) with the decimal point simply used to group the 3 digits, whereas in the US and Asia it can be read as 11 point three-six-two. Vice versa, the comma in a value of “11,362” stored in a data file can erroneously be interpreted by a machine as column delimiter, splitting the single value up in two separate values of 11 and 362. Thus, the decimal sign should be kept constant to make processing easy.

#### Avoid coding of missing values

Empty or non-entered values are typically used to indicate missing values. If a missing value needs to be distinguished from an empty value or a refused answer, use a consistent method and ensure it cannot be misinterpreted as a possible value. It is recommended to replace artificial codes such as “-99” with “NA” (not available)^25,28^.

#### Account for high or low measurement thresholds in measuring devices

If a machine reports a measurement above or below the measuring threshold, it should not be considered a missing value as this would significantly bias the data. Similarly, the high or low threshold should not be entered as a genuine value as this would introduce bias as well. Instead, the data should indicate if the value exceeds the measurable range and in which direction. Values exceeding thresholds need to be easily identifiable, such that an accidental usage of the threshold as actual value is avoided. Separate documentation should specify these thresholds. Depending on the analysis, the data can be handled by categorising values or imputation.

### Obtaining data from repositories

Frequently, researchers who analyse data are not responsible for collecting it and must rely on large repositories. While they may not be able to alter the collection process or structure, they can provide feedback to the collecting institute and request changes. These changes may include any of the aforementioned points, as well as repository-specific modifications. The latter aim to enable access to the same data regardless of time or user.

#### Document and standardize queries

Similar research questions or even the more specific data request may lead to different data queries that are used to extract data from data repositories despite the best efforts of defining them in a consistent manner either from researchers themselves or the repository staff. Therefore, queries should be standardized and documented to ensure that subsequent data requests can be performed in the same manner. To avoid discrepancies, it is preferable to base these queries on code rather than relying on generic descriptions. This not only ensures efficiency but also guarantees that researchers with the same data request and analysis obtain identical results as long as there are no changes in the repository itself.

#### Use versioning

Changes in the data itself can cause the same data request to produce different data output. This can occur due to new data being added or quality control measures. Therefore, it is important to not only know the time range of the collected data but also be aware of any changes in the data versioning. This becomes a critical issue when previous versions are unavailable or version numbering is absent. Optionally, for example when transferring data, it can be helpful to calculate MD5 file hashes and compare them between the data versions to confirm that they are identical.

#### Document used data

Document the time and source of the data received, regardless of whether the data provider guarantees the withdrawal of the same data at a later stage, and store it together with the received, unprocessed data. This is the only way to establish correct references or explain differences. Ideally, data sets used for re-analysis or conduction of meta-studies should be unambiguously identified using persistent DOIs. Since these identifiers also contain information about earlier or later versions of data sets from a continuing project, they inherently avoid a potential confusion when analyses are repeated at different time points.

### Processing the data

The processing stage is a critical part of data handling, where errors can be easily introduced and difficult to detect. While it is impossible to completely rule out mistakes, there are many ways to reduce their likelihood. Several of the guidelines presented here are standard in general software development, of which there are many, however, we focus on the most common and crucial issues for data handling and data analysis.

#### Document structure and requirements of pipeline

To ensure the processing pipeline can be run smoothly and the files can be located easily, it is important to provide clear instructions preferably in a separate file. Additionally, it is necessary to state the versions, dependencies, packages, parameters, and prerequisites. This will help keep the project organised and user-friendly for both yourself and other researchers. In general, incorporating semantic standards can enhance the interoperability of your pipeline, ensuring that data and processes are linked meaningfully across different platforms. This may involve standardizing data structures or applying controlled vocabularies, such as classifications for diseases^31^.

#### Use scripts

To ensure comprehensibility, objectivity, and reproducibility, it is recommended to avoid manual data processing. Instead, scripted analysis programs such as R, MATLAB, SAS, or Python should be used^25^. These programs may require more effort initially, but they are essential for making the data processing understandable. They allow for easy retracing of processing steps, changing the approach, and recovering initial values for different analyses. This differs from software that uses primarily a graphical interfaces, where users choose methods from dropdown menus, making it difficult or impossible to document and reuse steps^27^. Furthermore, the use of scripts avoids the creation of multiple versions of processed data. Scripts can be easily versioned or branched, for example, by using Git. In the end, even though convenient, all processed data sets should be disposable since the procedure how to create them from the raw data should be clear and the scripts should always produce the same result.

#### Structure and describe scripts

Adding a description at the beginning of any script and making the structure as modular as possible will be an essential element of keeping your processing understandable and reproducible^25^. It is important to follow coding guidelines and provide sufficient commentary and documentation on how to run the script. Additionally, removing obsolete code and cleaning up the script avoids confusion in the future and should be done before the final run generating the data.

#### Perform quality control of your data

Data should be monitored in a standardized manner to detect irregularities^26^. This is especially relevant for data collected over a longer period of time, as continuous monitoring during the collection process can help identify issues. It is important to check for duplicates, impossible values, outliers, trends, and differences with respect to meta values^32^. For instance, weight cannot be negative, and observations may be influenced by time and different experimenters, which could indicate a problem in the data quality. Therefore, it is necessary to document them. Additional datasets and literature can be consulted to determine acceptable and expected ranges.

Regarding processed data, small errors in the code can significantly impact the analysis. Structuring the code, adding comments, and making it modular can mitigate this risk. Nonetheless, it is still essential to have quality checks during processing and analysis and to apply common sense and domain knowledge.

#### Separate data management and analysis steps

At the end of the data processing, there should be a consistent and robust dataset, along with clear documentation on how to create it. Once prepared, the analysis of the processed data can begin. It is recommended to perform the analysis in a separate file that only requires the processed dataset files. This modularity helps to maintain a clear structure and provides an overview of the process.

#### Avoid repetition of code snippets or input

Making your code modular and reusing modules using functions or loops can help to avoid the need for correcting or changing the same thing multiple times in the script. Otherwise, inconsistencies may occur. Similarly, it is preferable to define input variables at the beginning rather than defining them multiple times or in unnoticeable places^25^. For example, if you need to convert temperature data to Celsius at various points in your script, it is preferable to have a simple function that does the job rather than writing out the calculation multiple times. If the target output changes from Fahrenheit to Celsius at any point, then the change only needs to be made at one point.

#### Use descriptive and clear variable names in your code

When defining variables in your script, use clear and descriptive variable names consisting of basic letters, numbers, and underscores. This also applies to function names. Employing good names makes your code easier to understand, adapt, and debug. A variable name “temp” does not convey any meaningful information, while “average_temperature” clearly indicates the purpose. A function that converts temperature to Celsius should rather be called “convert_temperature_to_celsius” instead of “calc” directly conveying its purpose.

#### Transform the data into a format that is easy to analyse

Converting data into a format that can be easily inserted into typical analysis functions can save time, prevent errors, and increase understandability. Tidy data is often a useful format, where each variable is a column and each row is an observation^33^. Typical examples of messy data are studies where a subject is measured multiple times and each measurement of the subject is given a separate column. It would be preferable to have a separate row for each measurement indicating the number of the measurements and the subject to which the measurement belongs. For more detailed guidelines on structuring tidy data, relevant publications can provide further insights. It is important to note that any reasons for missing values should be entered in a separate column, rather than in the column of measurements. But easy to analyse data also extends to other aspects mentioned before, such as not saving information by colouring cells or a consistent nomenclature. An example of suitable and unsuitable datasets can be found in the supplementary material and the Gitlab repository https://ascgitlab.helmholtz-munich.de/cf_statcon/GRDI.

#### Ensure traceability when merging datasets

The merging of datasets should be script-based and be based on identifiers unique across all data sources. This helps to prevent incorrect mapping and duplication of entries. Consider merging patient records from two hospitals. Merging by identifier is already better than merging by name, but it still needs to be ensured that an identifier in one hospital corresponds to the same person as the same identifier in the other hospital.

#### Record and report all changes in the data

Data changes may be necessary. To make changes, use a script and document the reasons for the changes. When making changes, use objective criteria rather than row numbers. For instance, if negative body weights are recorded, do not modify the numbers in the raw files. Instead, use processing and entries based on the criterion “weight < 0”.

## Conclusion

Research data can be easily altered, falsified, or rendered unusable, intentionally or unintentionally. This study focuses on practical data handling issues that directly affect analysis results and often receive little attention. It fills a critical gap in the reproducibility of the data handling process. This problem is particularly serious as it is rarely reported, despite previous publications discussing data accessibility. Results that are impacted by inadequate handling may not be immediately apparent and may only be partially revealed in reports of non-reproducibility.

The provided guidelines do not focus on the publication of data or scripts, as this aspect has already received considerable attention. Similarly, the main focus of this work is not on aspects such as interoperability and accessibility. While these are crucial considerations that can impact data handling, they are more aligned with higher-level data management practices. Existing guidelines and examples can be consulted for establishing such frameworks^6,31^. Instead, this publication offers guidance for researchers who wish to enhance the robustness, impact, and reproducibility of their research by raising awareness of data integrity and providing clear and practical guidance to ensure that it is considered at the planning stage of any project involving data and is maintained throughout. Furthermore, it encourages critical discussion about how inadequate data handling affects reproducibility, emphasizing that all stages of the data process are critical.

The proposed guidelines establish a standard and reference that enables researchers to depend on a certain level of quality and reproducibility of the data. This is important because some practices may be considered obvious by some, while others may not even consider them at all, leading to unclear assumptions.

Although researchers may encounter new challenges in handling data, the provided principles and awareness of the importance of robust research data can guide them. Adherence to the suggested guidelines is essential for fostering future data utilization, enabling the seamless reuse of data processing and analysis code, and enhancing effective communication with collaborators.

Future research should determine the impact of poor data handling on scientific output and how much non-reproducibility can be attributed to it. Additionally, scientific fields that require specific data handling should consider formulating more precise guidelines for their requirements.

## Supplementary information

GRDI checklist

Screenshots of data examples
