# Supplementary material for: Guidelines for Research Data Integrity (GRDI)
Source: Sci Data. 2025 Jan 17;12:95. doi: 10.1038/s41597-024-04312-x (PMC11739391; doi:10.1038/s41597-024-04312-x)
Supplement: Supplementary file 1 — GRDI checklist [file 41597_2024_4312_MOESM1_ESM.pdf]

GRDI: Guidelines for Research Data Integrity

This checklist offers key practices for maintaining data quality and reproducibility throughout your research. It spans planning, variable definition, data collection, and processing. For additional details and examples, visit https://ascgitlab.helmholtz-munich.de/cf_statcon/GRDI.

1. Defining the strategy

1.1 Plan study, data requirements, and analysis together
1.2 Write a data dictionary
1.3 Save your data in an accessible and general-purpose file format
1.4 Keep the raw data

2. Defining all variables

2.1 Avoid repetition
2.2 Avoid combining information
2.3 Use simple language
2.4 Give short but informative variable names
2.5 Make column names machine-readable
2.6 Record metadata
2.7 Use a suitable tool for data capture, management, and storage
2.8 Account for varying levels of measurement accuracy among entries

3 Defining the collection process

3.1 Use identifiers
3.2 Be concise but avoid ambiguity
3.3 Restrict data entry to possible values
3.4 Be consistent with nomenclature
3.5 Avoid coding of missing values
3.6 Account for high or low measurement thresholds in measuring devices

4 Obtaining data from repositories

4.1 Document and standardize queries
4.2 Use versioning
4.3 Document used data

5 Processing the data

5.1 Document structure and requirements of pipeline
5.2 Use scripts
5.3 Structure and describe scripts
5.4 Perform quality control of your data
5.5 Separate data management and analysis steps
5.6 Avoid repetition of code snippets or input
5.7 Use descriptive and clear variable names in your code
5.8 Transform the data into a format that is easy to analyse
5.9 Ensure traceability when merging datasets
5.10 Record and report all changes in the data
