# Supplementary material for: Guidelines for Research Data Integrity (GRDI)
Source: Sci Data. 2025 Jan 17;12:95. doi: 10.1038/s41597-024-04312-x (PMC11739391; doi:10.1038/s41597-024-04312-x)
Supplement: Supplementary file 2 — Screenshots of data examples [file 41597_2024_4312_MOESM2_ESM.pdf]

GRDI: Guidelines for Research Data Integrity - Examples

GRDI provides essential guidance for maintaining data quality and reproducibility throughout the research process. The following two screenshots illustrate some of the common pitfalls in data formatting and contrast them with a more optimal approach. For further details, please refer to https://ascgitlab.helmholtz-munich.de/cf_statcon/GRDI.

Diagnosis of diabetes						
Male or Female?	diab	BMI	Hemoglobin measured in g/dL			
F	y	65/1.65^2	13			Type 1
Female	yes	76/1.71^2	15			Type 2
male	no	82/1.76^2	14			
Male	y	69/1.61^2	9999			
female	no	79/1.73^2	12			
male	no	67/1.69^2	11			
Female	n	72/1.72^2	unknown			
m	yes	77/1.87^2	14			
female	yes	68/1.74^2	16			
male	no	84/1.81^2	10			
f	y	69/1.71^2	12			
male	yes	78/1.78^2	16			

Supplementary Figure S1: Bad example of how data can be formatted and saved as xlsx.

ID	sex	diabetes	diabetes_type	weight_kg	height_m	hemoglobin_gdL
1	female	yes	1	65	1.65	13
2	female	yes	2	76	1.71	15
3	male	no	NA	82	1.76	14
4	male	yes	1	69	1.61	NA
5	female	no	NA	79	1.73	12
6	male	no	NA	67	1.69	11
7	female	no	NA	72	1.72	NA
8	male	yes	1	77	1.87	14
9	female	yes	2	68	1.74	16
10	male	no	NA	84	1.81	10
11	female	yes	2	69	1.71	12
12	male	yes	1	78	1.78	16

Supplementary Figure S2: Good example of data formatting as csv.
